# Fiber-Optic Raman Sensor for Early Dental Caries Detection: Performance Evaluation and Robustness to Probe Positioning

**DOI:** 10.3390/bios16060334

**Published:** 2026-06-11

**Authors:** Sofia Pessanha, João Miguel Silveira, Paulo Ribeiro, António Mata, Valentina Vassilenko, Sofia Barbosa

**Affiliations:** 1 NOVA School of Science and Technology, Campus Caparica, 2829-516 Caparica, Portugal; pfr@fct.unl.pt (P.R.); vv@fct.unl.pt (V.V.); svtb@fct.unl.pt (S.B.); 2 LIBPhys, LA-REAL, Campus Caparica, 2829-516 Caparica, Portugal; joao.silveira@fmd.ulisboa.pt (J.M.S.); antonio.mata@fmd.ulisboa.pt (A.M.); 3 Faculty of Dentistry, University of Lisbon, R. Professora Teresa Ambrósio, 1600-277 Lisbon, Portugal; 4 GeoBioTec (UNL)—GeoBiosciences, GeoTechnologies and GeoEngineering, Department of Earth Sciences, Campus FCT-UNL, 2829-516 Caparica, Portugal

**Keywords:** confocal Raman microscopy, fiber-optic coupled sensor, dentistry, diagnosis

## Abstract

Early detection of dental caries remains a significant clinical challenge, as conventional diagnostic methods lack sensitivity for incipient lesions. Raman spectroscopy offers high chemical specificity for enamel characterization; however, clinical translation is hindered by the complexity of conventional polarized confocal systems. In this work, we present a Raman-based fiber-optic sensing approach for the detection and classification of dental enamel conditions, including sound, affected, and carious tissues. A custom fiber-optic probe was developed for remote measurements and evaluated against a reference polarized confocal Raman system. In addition to spectral discrimination, key factors affecting sensing performance were investigated, including spatial variability across enamel surfaces and angular sensitivity due to probe misalignment. Raman-derived features (carbonate-to-phosphate ratio, phosphate peak intensity, position, and bandwidth) were analyzed using a multinomial logistic regression classifier. The fiber-optic sensor achieved an overall classification accuracy of 73% (F1-scores: 0.55 sound, 0.63 affected, 0.9 carious), confirmed by leave-one-tooth-out cross-validation. Probe misalignment studies revealed robustness up to 10° angular deviation. These results demonstrate that a simplified non-polarized fiber-optic Raman system provides competitive diagnostic performance and clinically relevant robustness, supporting its development as a point-of-care sensing platform for early dental caries detection.

## 1. Introduction

Traditionally, the loss of mineral content in teeth was believed to be permanent; however, in recent decades, a paradigm shift has occurred in dental care, where a more preventive and conservative approach is being pursued. There are, nowadays, several products advocating for the restoration of lost mineral [1,2] and/or strengthening of the hydroxyapatite lattice [3]. However, for these products to be used efficiently, the diagnosis must occur as early as possible in the progression of the disease and, ideally, these diagnosis methods should be fast, accurate, and as inexpensive as possible.

The most common clinical diagnostic method for hypomineralized enamel is still visual detection or probing with a dental explorer, which are characterized by their qualitative nature. A common evaluation can be performed according to the International Caries Detection and Assessment System (ICDAS), highly dependent on clinician expertise, leading to variability in detection rates. These methods are commonly aided by digital radiography, providing a greyscale image of the tooth according to the specific attenuation of the radiation in the different tissues. However, the yielded results are sometimes deficient when dealing with incipient or occlusal carious tissues [4]. Other methods, based on changes in the optical properties of enamel, have been developed, taking advantage of the porosity induced in the demineralized areas that will increase the scattering of incident radiation. In contrast, sound enamel comprises densely packed crystals, producing an almost transparent structure. Within these methods, there are devices for fiber-optic transillumination (FOTI), using either a halogen lamp or near-infrared probe to inspect the tooth perpendicularly to the lingual and vestibular surfaces and interpretation of the transmittance of light through the tooth [5]. Other diagnostic assistance methods are based on fluorescence of enamel, grounded on the principle that autofluorescence of the tooth changes as the mineral content is altered. Some devices make use of the differentiated autofluorescence of sound and demineralized enamel when radiation of a certain wavelength interacts with the tooth. These devices, namely Quantitative Light-induced Fluorescence-QLF™ Pro camera (Inspektor, Bussum, The Netherlands) [6], DIAGNOdent^TM^ (KaVo, Biberach, Germany) [7], or VistaCam (Dürr Dental, Bietigheim-Bissingen, Germany) [8], are calibrated to deliver a quantitative evaluation of the tooth. However, appropriate calibration is difficult since the intensities depend also on the positioning of the probe and the shape of the tooth [9].

Staining, calculus, and organic material may also lead to some false positives and false negatives, so the sensitivity and specificity of each of these diagnostic sensors are less than optimal [10].

Raman scattering is an interaction of radiation with matter used for the inspection of vibrational modes of materials. When dealing with human enamel, mainly composed of carbonated hydroxyapatite, several molecular vibrational modes are Raman-active, with some of them highly anisotropic and sensitive to the crystalline orientation and organization of hydroxyapatite crystals. We can take advantage of this property to diagnose the crystallinity and, hence, the mineralization degree of enamel. Raman scattering has been used, in vitro, in early caries determination and in the diagnostics of other causes of enamel demineralization resulting from daily activities and pharmaceutical routines [3,11,12,13,14,15,16]. Also, the background of the Raman spectra carries information regarding the fluorescent compounds in the enamel which appear in the caries lesions and can aid the diagnostics [17].

Making use of polarized Raman spectroscopy and determining the depolarization ratio of the symmetric stretching band of phosphate in the enamel structure carious lesions [18,19] can be recognized, and the effects of fluorinated tooth bleaching products can be evaluated [13]. Similarly, this approach can be used to investigate the protective suitability of a dental fluorinated varnish [15] and the efficacy of different protocols of application of remineralizing tooth mousse based on CPP-ACP (casein phosphopeptides and amorphous phosphate complex) [3,14]. There have also been attempts to perform ex vivo measurements of dental enamel using remote probe Raman analysis [20,21] for the early diagnosis of demineralization.

Complementary surface-enhanced Raman scattering (SERS) approaches, reviewed recently [22,23], have demonstrated exceptional sensitivity in biological sensing contexts; however, their application to intact enamel surfaces remains challenging, motivating continued development of conventional fiber-optic Raman platforms.

The use of fiber optics enables remote tissue analysis, but it presents several challenges and limitations when developing an in vivo diagnostic device [24]. These challenges typically include concerns related to the signal-to-noise ratio, balancing measurement time with accuracy, light scattering caused by imperfections in the fiber, potential errors from probe positioning, and depolarization effects induced by the optical fiber itself [25,26]. However, using polarized Raman spectroscopy, in vivo, poses significant challenges, primarily due to the difficulty in controlling the polarization of both the incident and collected signals. Additionally, maintaining polarization through optical fibers is problematic because of stress-induced birefringence and depolarization effects [27].

Although Raman spectroscopy has demonstrated strong potential for early caries detection, existing studies on fiber-optic implementations remain limited in three key aspects: the lack of direct comparison with a gold-standard polarized Raman microscopy system using matched samples and protocols; the insufficient systematic evaluation of probe positioning effects—both spatial variability across the enamel surface and angular sensitivity to probe tilt; and the limited assessment of diagnostic performance across three enamel conditions using multivariate statistical frameworks. These limitations hinder the translation of Raman methodologies into clinical practice.

From a sensing perspective, an effective diagnostic system must not only provide sufficient chemical contrast but also demonstrate robustness to measurement variability and operational simplicity. In this context, Raman spectroscopy offers significant potential as an optical sensing modality; however, its implementation in clinically compatible platforms remains limited. This work addresses this gap by evaluating a fiber-optic Raman probe as a diagnostic sensor, focusing on performance metrics and robustness under realistic measurement conditions.

This work addresses these gaps by directly comparing a fiber-optic Raman sensor [28] with polarized confocal Raman microscopy, systematically evaluating spatial and angular probe positioning effects, and assessing diagnostic performance using multivariate statistical modeling. The performance of each approach was quantitatively assessed using standard diagnostic metrics, including sensitivity, specificity, and accuracy. The purpose of this work is to further understand if the fiber-optic Raman system, despite its simpler configuration, achieves competitive diagnostic performance, reinforcing its potential for future integration into routine dental diagnostics and chairside applications.

## 2. Methods

### 2.1. Sample Description

A total of 30 (10 sound and 20 carious, non-restored) human teeth (premolar and molar) previously extracted for periodontal or orthodontic reasons and preserved in a 0.5% (*w*/*w*) chloramine T3—H_2_O solution, at 4 °C, were selected. The diagnosis and caries recognition were performed by experts in the research team using visual–tactile inspection. Moreover, the sound specimens were carefully observed under a stereomicroscope (Meiji Techno EMZ 8RT, Saitama, Japan) to exclude the presence of lesions. Samples were stored in properly identified storage vials in a new chloramine solution until the beginning of the measurements. In this work, three experimental groups of human dental enamel samples were analyzed. These were Group A (n = 10): sound enamel from caries-free teeth confirmed by visual–tactile inspection and stereomicroscopy; Group B (n = 10): affected enamel, defined as vestibular/lingual enamel regions from carious teeth showing no visual or tactile signs of demineralization at the measurement site (included to probe whether enamel from a caries-bearing tooth exhibits spectroscopic differences from fully sound enamel, potentially reflecting early mineral changes beyond the macroscopic lesion boundary); and Group C (n = 10): carious enamel sampled at cavity edges or white-spot regions confirmed by expert clinical assessment.

During the experiment, all examined teeth samples were not brushed, treated, or kept in human saliva, or other solutions, to maintain the same experimental conditions; a dry environment was utilized. There was no significant dehydration of the sample enamel surface. This was avoided since all Raman measurements, for both of the approaches, were performed within 15 days.

### 2.2. Raman Measurements

#### 2.2.1. Polarized Raman Confocal Microscope

Polarized Raman spectra were obtained using an XploRA Confocal Microscope (Horiba, Palaiseau, France) with a 785 nm laser. Using an entrance slit of 200 μm, and a confocal hole of 300 μm, the scattered light collected by the objective was dispersed onto the air-cooled CCD array of an Andor iDus detector (Oxford Instruments, Belfast, Northern Ireland) with a 1200 lines/mm grating. This way, the spectral range investigated was from 400 cm^−1^ to 1500 cm^−1^ with spectral resolution of 4 cm^−1^. A 10× objective (N.A. = 0.9) was used to focus on the surface of the enamel, as well as a 50% neutral density filter rendering an incident power on the sample of 9.0 ± 0.4 mW (lasercheck^®^, Edmund optics, Barrington, NJ, USA). Each spectrum was obtained by three accumulations of 15 s each. In order to determine the depolarization ratio (ρ959) of the symmetric stretching band of phosphate (ν1~959 cm^−1^), in each spot, three spectra were recorded, without polarization and with cross and parallel polarization to the polarization of the incident laser [10]. Also, for a different approach into spectra evaluation, the profile of the symmetric stretching of phosphate was further inspected, comprising the wavenumber of Raman shift, the Full Width at Half Maximum (FWHM), and the evaluation of the b-type carbonate substitution by comparing the carbonate-to-phosphate ratio [29,30,31]. For each sample, 10 spots were probed.

#### 2.2.2. Remote Fiber-Optic Sensor

**Figure 1:**
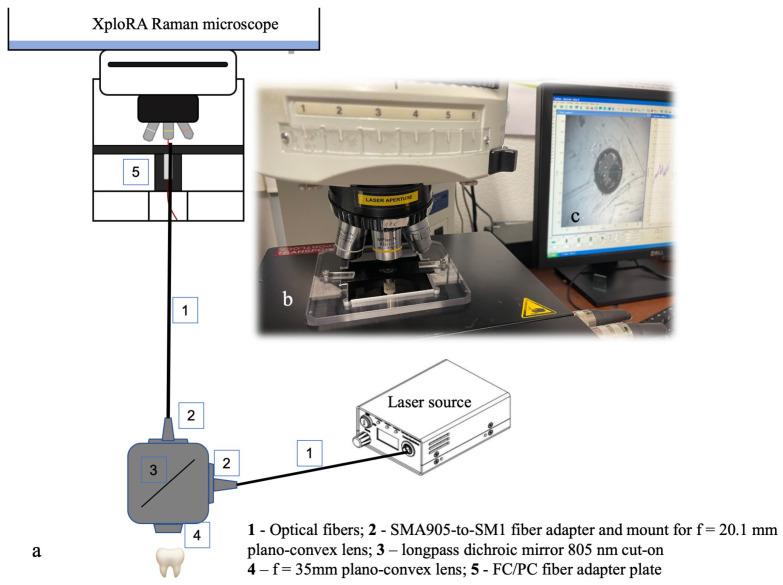
(**a**) Scheme of the remote probe setup, with identification of the different components and adapters. (**b**) Detail photograph of the FC/PC fiber adapter mounted in the microscope plate and (**c**) the focused image of the optical fiber entrance. Figures are not to scale.

The fiber-optic sensor (Figure 1) was developed and assembled within the research group; further details can be found in Pessanha et al.’s study [28]. The laser power of the external 785 nm laser (CNI, Changchun New Industries Optoelectronics Technology Co. Ltd., Changchun, China) was set to 80% of the maximum power, rendering a power on the sample of 90 ± 3 mW (lasercheck^®^, Edmund optics). The scattered radiation was then collected by the probe lens (LA4647, Thorlabs, Bergkirchen, Germany) and steered using a low-OH multi-mode optical fiber (FT200EMT 200 μm core diameter. N.A. = 0.39 Thorlabs, Germany) conducted through the exiting optical fiber onto the XploRA microscope stage fixed with an FC/PC adapter (Horiba, France) and collected using the microscope 10× lens (N.A. = 0.25). Using an entrance slit of 200 μm, and a confocal hole of 500 μm, the scattered light collected by the objective was dispersed onto the air-cooled CCD detector Andor iDus (Horiba, France) with a 1200 lines/mm grating. This way, the spectral range investigated was from 400 cm^−1^ to 1500 cm^−1^, with a spectral resolution of 10 cm^−1^. Energy calibration was performed using a diamond sample, and each spectrum was obtained by three accumulations of 15 s each. For each sample, 10 spots were probed. A long-pass edge filter (>800 nm) was placed at the collection end to suppress Rayleigh scatter and fiber background before injection into the spectrometer; a short-pass filter at the excitation end suppressed laser ASE. The complete probe parameters are as follows: fiber FT200EMT (Thorlabs), 200 µm core, NA = 0.39; laser 785 nm CW (CNI), 90 ± 3 mW at sample; collection lens LA4647 (Thorlabs), f = 25.4 mm; spectrometer 10× objective (NA = 0.25), slit 200 µm, confocal hole 500 µm, 1200 L/mm grating, spectral resolution 10 cm^−1^.

#### 2.2.3. Spectra Deconvolution, Preprocessing and Fitting

Data processing and analysis were performed in the interface Visual Studio Code^®^ (Microsoft, Redmont, WA, USA) version (1.96.0) using Python version (3.12.2). File management and batch processing were automated using the os, glob, and re libraries. Tabular and numerical data were handled with Pandas and NumPy. Preprocessing included smoothing of the signal using the Savitzky–Golay filter (savgol_filter) and correction of baseline drifts using the modified polynomial (modpoly) algorithm from the Pybaselines package. Nonlinear curve fitting was conducted with SciPy’s curve_fit function, and peak shapes were modeled with the Voigt profile (scipy.special.voigt_profile). Data visualization was performed using Matplotlib, and logging was used throughout to ensure reproducibility of preprocessing steps.

The first step was to apply the Savitzky–Golay filter (savgol_filter) to smooth the spectra and remove uncorrelated noise. The filter was applied with a window size of five data points and fitting a third-order polynomial to this window. The second step consisted of background removal, caused by fluorescence of the sample and baseline of the CCD. Background was removed by the modified polynomial fitting algorithm (modpoly) from the Pybaselines package. The output files were then analyzed and the peaks deconvoluted using Gaussian profiles. For the non-polarized spectra, this procedure was applied to the phosphate (~959 cm^−1^) and carbonate peaks (~1070 cm^−1^), and the amplitude, centroid of peak, and Full Width at Half Maximum were obtained. For the polarized spectra, only the amplitude of the phosphate peak (~959 cm^−1^) was gauged and compared to determine the depolarization ratio [11].

## 3. Evaluation of the Influence of the Positioning of the Sensing Probe

**Figure 2:**
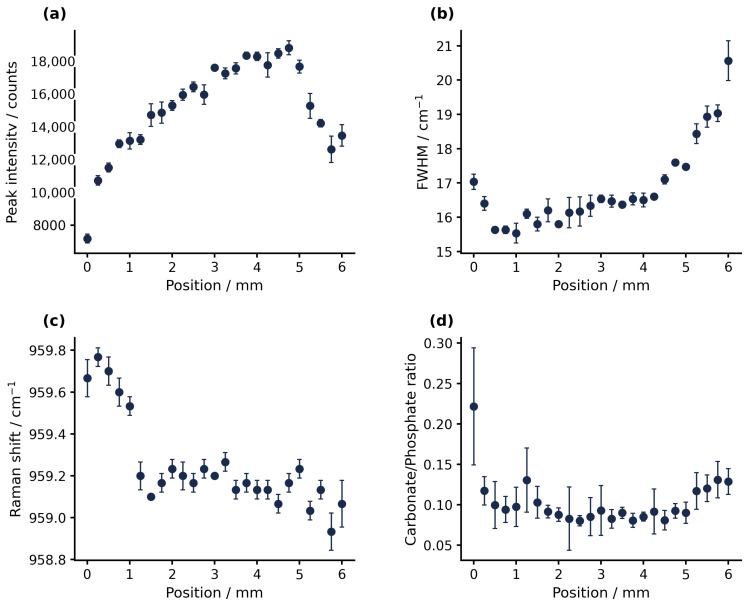
Spatial variation in the Raman spectral parameters across a tooth, highlighting distinct regions of chemical and structural composition. (**a**) Phosphate peak intensity, (**b**) Phosphate peak Full Width at Half Maximum, (**c**) Phosphate Raman shift wavenumber, and (**d**) Carbonate to phosphate intensity ratio.

**Figure 3:**
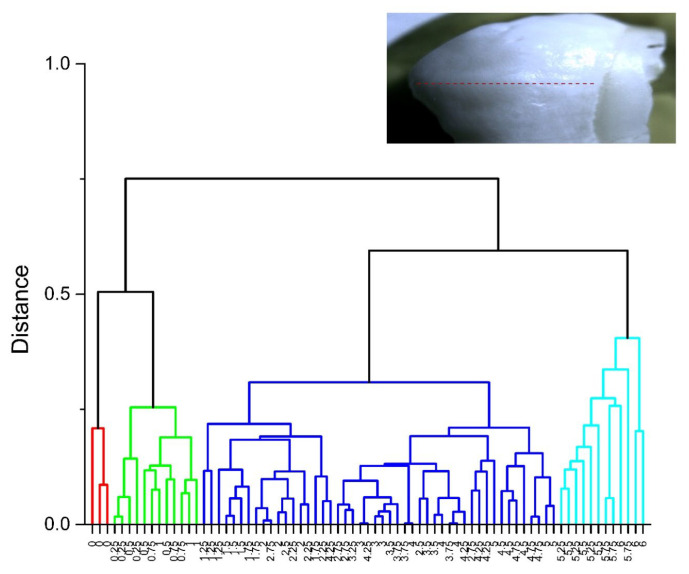
Hierarchical cluster analysis identifying four distinct clusters, corresponding to spatial regions with similar spectral characteristics. On the xx axis are the several probed points from the cusp to the cervical region of the tooth.

The orientation of the hydroxyapatite crystals is different throughout the enamel surface, and this orientation can influence the parameters we obtain from the Raman spectrum. This influence in polarized Raman microscopy was previously studied by Pezzotti et al. [19]. In order to gauge this influence and source of uncertainty in the results for the remote probe, we measured the Raman profile in a sound specimen of a molar tooth. We measured from the tooth cusp down to the cervical region by placing the sample in a stage with a microtome screw and measuring in steps of 250 μm, collecting three spectra in each position. Special attention was paid to adjust the distance from the probe lens to the sample to adjust to the curvature of the tooth. Figure 2 and Figure 3 illustrate the spatial variation in the Raman spectral parameters across a tooth, highlighting distinct regions of chemical and structural composition.

Figure 2a shows the phosphate peak intensity, which increases from 0 to 4 mm and decreases thereafter, reflecting changes in mineral content. Figure 2b depicts the Full Width at Half Maximum (FWHM), indicating a gradual increase in peak broadening, suggestive of higher structural disorder. Figure 2c,d reveal shifts in the phosphate peak wavenumber and in the carbonate-to-phosphate ratio, representing subtle compositional variations. Figure 3 presents the hierarchical cluster analysis identifying four distinct clusters, corresponding to spatial regions with similar spectral characteristics. The inset image provides spatial context, showing the analysis line along the tooth.

As can be seen, four distinct clusters are formed: the point corresponding to the cusp (in red); the cuspidal region up until 1 mm (in green); the cervical region of the tooth, from 5 mm onwards (in light blue) and the larger cluster; and the central region of the tooth, between 1.25 and 5 mm (in dark blue). Therefore, to minimize the influence of crystallographic orientation on the Raman signal intensity, particularly in molar teeth, where this deviation is more pronounced in the occlusal region, we avoided collecting data from occlusal surfaces [18] as well as from the cervical region. For the sound enamel group (Group A), measurements were performed on vestibular/lingual surfaces of molars and premolars. In the case of carious lesions (Group C), the measurements were concentrated at the cavity edges or white-spot regions in the vestibular/lingual area. For the affected enamel group (Group B), the opposite surface of the caries was selected for measurements also aiming at the vestibular/lingual area. Particularly, the same regions were used for measurements with both Raman systems to ensure consistency.

Moreover, the orientation of the probe, designed to be used in a backscattering geometry, might also influence the extracted parameters. This way, several measurements were performed in the same spot, starting from the ideal position and then gradually moving the probe 1º at a time using a goniometer. Three measurements were performed in each spot.

**Figure 4:**
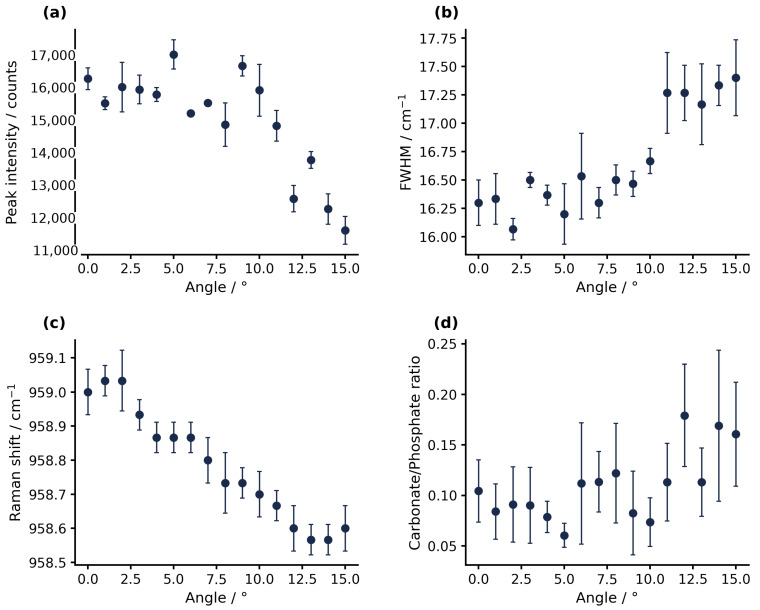
Evolution of Raman spectral parameters with laser incidence angle and the results of k-means clustering analysis. (**a**) Phosphate peak intensity, (**b**) Phosphate peak Full Width at Half Maximum, (**c**) Phosphate Raman shift wavenumber, and (**d**) Carbonate to phosphate intensity ratio.

**Figure 5:**
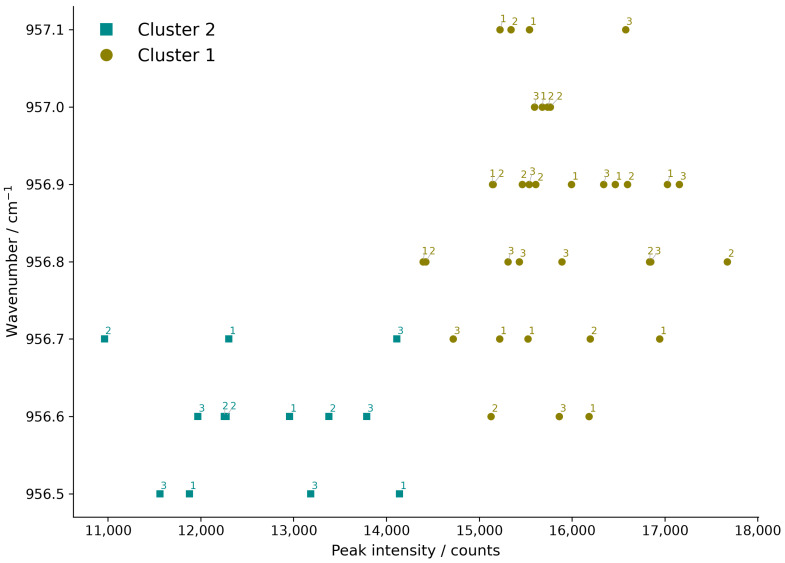
The k-means clustering results separating the data into two distinct clusters based on peak intensity and wavenumber. Numbers correspond to the probed angle.

Figure 4 and Figure 5 present the evolution of Raman spectral parameters with laser incidence angle and the results of k-means clustering analysis. Figure 4a shows the phosphate peak intensity, which decreases markedly as the angle increases beyond 10°. Figure 4b illustrates the Full Width at Half Maximum (FWHM), which broadens progressively with increasing angles, reflecting heightened structural heterogeneity. Figure 4c displays the wavenumber shift in the phosphate peak, with a clear decline as the angle increases, indicating angular sensitivity in the Raman response. Figure 4d depicts the carbonate-to-phosphate ratio, indicating less sensitivity for probe orientation. Figure 5 presents the k-means clustering results separating the data into two distinct clusters based on peak intensity and wavenumber. Cluster 1 corresponds to lower angles, while Cluster 2 dominates higher angles, suggesting a critical threshold for maintaining spectral integrity, θ < 10°. This value is quite large, suggesting that when using the three parameters combined, small tilting of the probe will not affect the characterization and diagnosis.

Short-term repeatability of the fiber-optic probe, assessed from three replicate spectra at angular position 0° (optimal alignment), yielded RSD < 3% for phosphate peak intensity and <2% for the carbonate-to-phosphate ratio. Inter-session reproducibility was not formally assessed in this study; all measurements were performed in a single session per tooth with energy calibration making use of a diamond reference. Day-to-day reproducibility represents an important aspect to be validated in future work.

## 4. Statistical Analysis

Statistical evaluation was performed using dedicated python scripts based on the scipy.stats and scikit.posthocs libraries. Normality was evaluated using the Shapiro–Wilk test followed by the Kruskal–Wallis H-test, as all parameters present a non-normal distribution; subsequently, Dunn’s test for significant differences in non-normal distributions was performed to analyze differences between the groups. A significance level of *p* = 0.05 was considered. Moreover, a logistic regression model [32] was applied in order to predict sound, affected, and carious enamel through the parameters derived from Raman spectra, using a 70–30% training size–test size ratio. The accuracy, sensitivity, and specificity were calculated by the logistic analysis with a significance level of *p* = 0.05. In addition to accuracy, sensitivity, and specificity, the F1-score was used to evaluate classification performance. The F1-score, defined as the harmonic mean of precision and recall, provides a balanced metric particularly suitable for datasets with class imbalance. To address the potential instability of a single train–test split, leave-one-tooth-out cross-validation was additionally performed. In each fold, all spectra from one tooth were withheld as the test set, and the remainder were used for training.

## 5. Results and Discussion

**Figure 6:**
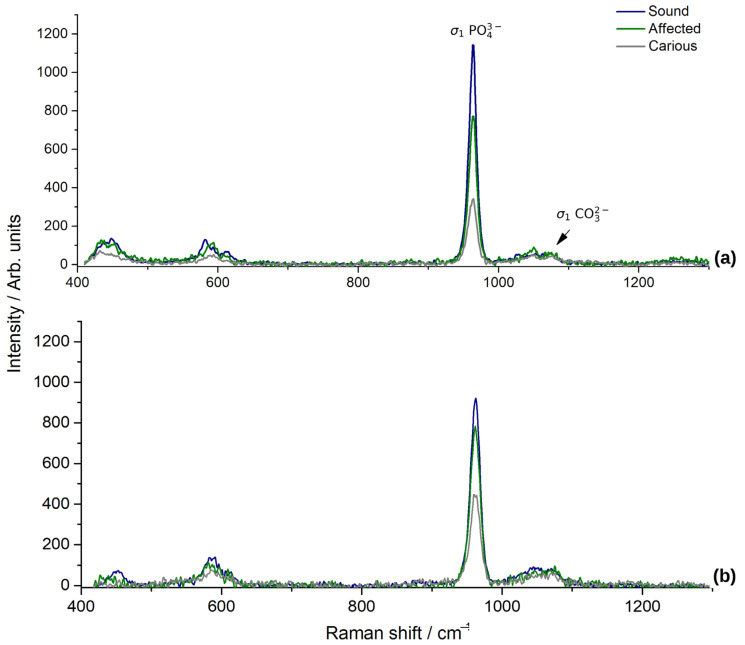
Examples of baseline corrected spectra of sound, affected, and carious enamel obtained with the XploRA benchtop microscope (Horiba, Palaiseau, France) (**a**) and with the remote probe (**b**). Highlighted are the bands corresponding to the symmetric stretching of phosphate and b-type carbonate.

**Figure 7:**
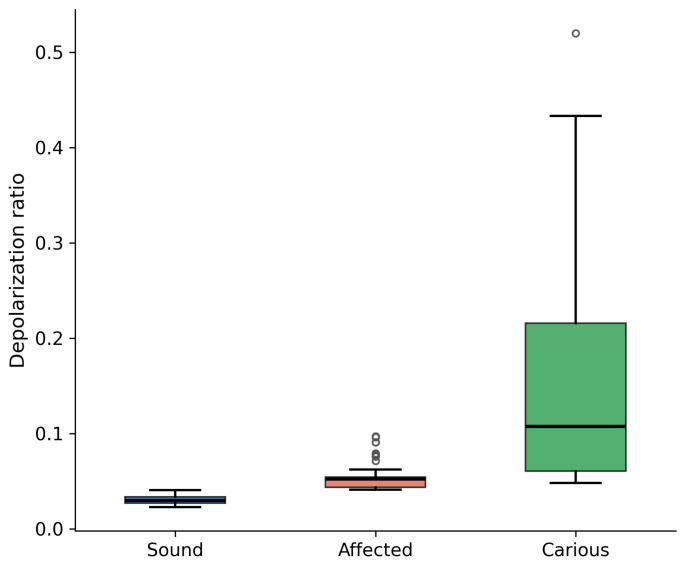
Comparison of the parameters extracted from the Raman spectra acquired with polarized microscope.

**Figure 8:**
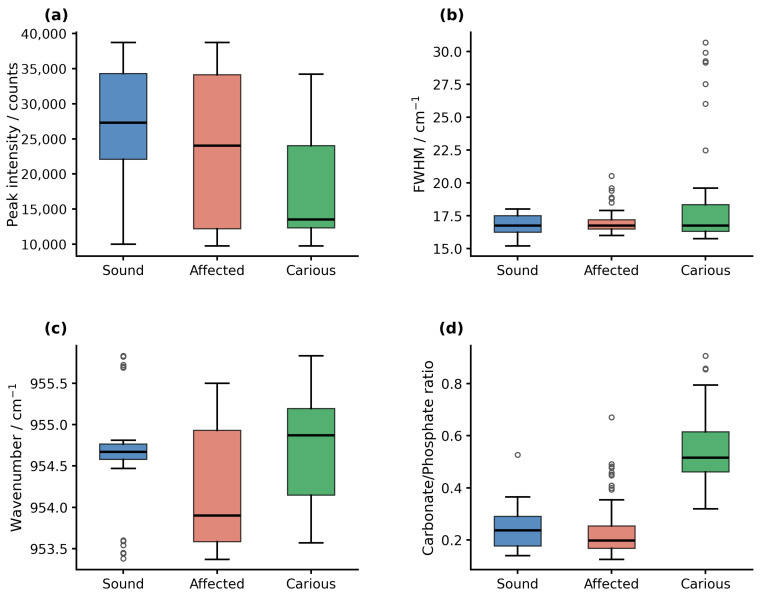
Comparison of the parameters extracted from the Raman spectra acquired with remote probe. (**a**) Phosphate peak intensity, (**b**) Phosphate peak Full Width at Half Maximum, (**c**) Phosphate peak wavenumber, and (**d**) Carbonate to phosphate intensity ratio.

Figure 6 presents representative spectra obtained with the benchtop microscope and with the remote probe for different substrates of enamel, namely sound, affected, and carious, while Figure 7 and Figure 8 present the box-plot charts for the comparison of the parameters extracted from the Raman spectra using a confocal microscope and the remote probe, respectively.

As can be seen in Figure 7, and comparing the depolarization ratio the three sample groups, this parameter increases from sound (mean = 0.031 ± 0.005) to affected (mean = 0.06 ± 0.02) to carious enamel (mean = 0.10 ± 0.01). The increasing behavior has already been determined in previous works by Buchwald et al. [33] and Ko et al. [10]. The Kruskal–Wallis H-test confirmed highly significant differences among the three groups (*p* < 0.001). Subsequent Dunn’s post hoc comparisons demonstrated strong statistical separation between all pairs: sound vs. affected (*p* < 0.001), sound vs. carious (*p* < 0.001), and affected vs. carious (*p* < 0.001).

This is also shown in the performance summary in Table 1, with an overall accuracy of 84% and F1-score of 0.99 (sound), 0.76 (affected), and 0.66 (carious).

This confirms the depolarization ratio as a robust discriminator across all tested tissue conditions.

Regarding the use of the remote probe and the comparison of four extracted parameters, Figure 8 shows the comparison of the box-plot charts obtained for the average values for each type of enamel condition.

Of the four Raman parameters investigated, only the carbonate-to-phosphate ratio showed statistical separation comparable to the depolarization ratio. While no significant differences were found between sound and affected enamel considering this ratio (*p* = 0.06), this parameter demonstrated extremely significant differences in Dunn’s post hoc tests when comparing carious enamel to both sound (*p* < 0.001) and affected enamel (*p* < 0.001). The increased substitution of phosphate by b-type carbonate has already been attributed to the development of carious lesions [28]. Similarly, phosphate peak intensity showed significant differences in distinguishing between sound and affected (*p* = 0.008) and sound and carious (*p* < 0.001) and affected carious enamel (*p* = 0.04), while phosphate peak wavenumber presented significant differences between sound and carious (*p* = 0.003) and affected and carious enamel (*p* = 0.002). In contrast, the FWHM parameter showed no significant difference across the groups (*p* = 0.48), suggesting minimal diagnostic contribution for these samples. The lack of statistical significance in FWHM suggests that structural disorder alone is not a reliable discriminator in early-stage lesions, likely because carbonate substitution precedes significant lattice disruption, while phosphate peak intensity showed significant differences between the three studied groups.

A second multinomial logistic regression model was constructed using these four parameters, and this approach resulted in a significantly improved overall test accuracy of 73%. The corresponding F1-scores were 0.55 for sound enamel, 0.63 for affected enamel, and 0.90 for carious enamel, reflecting a balanced trade-off between precision and recall across all classes. Notably, the higher F1-score observed for carious enamel indicates improved robustness in distinguishing this condition despite overlapping spectral features.

The lower accuracy observed for the fiber-optic probe (73% vs. 84%) can be attributed to several factors, including reduced spectral resolution (10 cm^−1^ vs. 4 cm^−1^), loss of polarization information, and increased sensitivity to probe positioning. Nevertheless, this reduction is offset by the significant gain in clinical applicability, as the sensing probe enables remote, flexible, and potentially in vivo measurements. Regarding translation to in vivo conditions, several factors deserve consideration. The salivary pellicle introduces organic Raman/fluorescence backgrounds, but the modified polynomial baseline correction handles such broad contributions, and the narrow spectral features used for classification are expected to remain discriminable. Surface contamination from plaque or calculus can be managed by standard pre-measurement air-drying and appropriate evaluation by the professional expert. Motion artifacts are partially mitigated by the demonstrated 10° angular tolerance; shorter acquisition windows and real-time signal quality monitoring will be explored in future in vivo studies.

The performance of the fiber-optic system demonstrates key characteristics of a practical optical sensor, including robustness to probe misalignment and tolerance to spatial variability across the enamel surface. The lower classification accuracy of the fiber-optic probe reflects inherent trade-offs: reduced spectral resolution, absence of polarization-resolved measurements, and greater sensitivity to probe positioning. These are offset by practical advantages such as accessibility to all tooth surfaces in oral cavity geometry; elimination of polarization control, which is technically impractical through flexible fibers; and substantially reduced cost and operational complexity. A 73% accuracy with balanced F1-scores across three enamel conditions compares favorably with commercial fluorescence-based devices (DIAGNOdent sensitivity ~60–70% [4]) while providing molecular-level chemical specificity.

## 6. Conclusions

This work demonstrates that a fiber-optic Raman system can operate as a reliable optical sensor for early dental caries detection. Despite reduced spectral resolution and the absence of polarization control, the system achieves robust classification performance and maintains tolerance to probe positioning variability. These characteristics are essential for practical implementation and support the development of Raman-based sensing platforms for non-invasive, real-time dental diagnostics.

Prior to any in vivo application, a rigorous laser safety evaluation is required. With the current probe configuration (785 nm, 90 mW at sample, estimated spot diameter ~200 µm), the irradiance at the enamel surface is approximately 2.9 W/cm^2^. While this exceeds the IEC 60825-1 MPE for extended soft-tissue exposure at 785 nm (~0.2–0.4 W/cm^2^), enamel absorption at this wavelength is substantially lower (~0.01 cm^−1^ [34,35]), meaning most incident radiation scatters elastically rather than being absorbed thermally. Literature data show that enamel withstands temperatures up to 200 °C for several minutes without structural damage [36], providing a significant thermal safety margin. A complete assessment—including thermal modeling, temperature measurements, and formal Maximum Permissible Exposure calculations—will be performed prior to any in vivo implementation.

Importantly, the use of a non-polarized Raman methodology greatly simplifies the technological setup, making it more feasible for clinical translation. The elimination of the need to control the polarization of both the incident and collected signals reduces the complexity and cost of the equipment, facilitating easier integration into dental practice. This simplification can lead to broader adoption and more widespread use of Raman-based diagnostics, enhancing the early detection and management of dental caries in a routine clinical setting. These findings pave the way for further development and clinical integration of Raman-based diagnostic sensors, offering a promising alternative to traditional methods for early and accurate caries detection.

By addressing key practical constraints such as probe positioning and system simplicity, this work provides a critical step toward the clinical translation of Raman-based dental diagnostics.

## Figures and Tables

**Table 1:** Performance summary regarding sensitivity, specificity, and precision when comparing A—sound enamel; B—affected enamel; and C—carious enamel.

Class	Sensitivity		Specificity		Precision	
	Benchtop System	Remote Probe	Benchtop System	Remote Probe	Benchtop System	Remote Probe
A—Sound	100%	51%	97%	88%	97%	60%
B—Affected	84%	66%	85%	75%	70%	61%
C—Carious	56%	90%	95%	93%	79%	89%

## Data Availability

Data will be available upon reasonable request.
